# Performing Permanent Distal Middle Cerebral with Common Carotid Artery Occlusion in Aged Rats to Study Cortical Ischemia with Sustained Disability

**DOI:** 10.3791/53106

**Published:** 2016-02-23

**Authors:** Christina Wayman, Denise A. Duricki, Lisa A. Roy, Barbara Haenzi, Shi-Yen Tsai, Gwendolyn Kartje, John S. Beech, Diana Cash, Lawrence Moon

**Affiliations:** ^1^Wolfson Centre for Age-Related Diseases, King's College London, University of London; ^2^Department of Neuroimaging, James Black Centre, Institute of Psychiatry, King's College London, University of London; ^3^Institute of Neuroscience and Psychology, Wellcome Surgical Institute, College of Medical, Veterinary and Life Sciences, University of Glasgow, Glasgow; ^4^Research Service, Edward Hines Jr. VA Hospital; ^5^Neurology Service, Edward Hines Jr. VA Hospital; ^6^Department of Molecular Pharmacology and Therapeutics, Neuroscience Research Institute, Loyola University Chicago; ^7^Department of Oncology, The Gray Institute for Radiation, Oncology and Biology, University of Oxford

**Keywords:** Medicine, Issue 108, Distal middle cerebral artery occlusion, MCAO, stroke, surgery, brain ischemia, elderly rats, sustained deficit, coagulation, sample size calculations

## Abstract

Stroke typically occurs in elderly people with a range of comorbidities including carotid (or other arterial) atherosclerosis, high blood pressure, obesity and diabetes. Accordingly, when evaluating therapies for stroke in animals, it is important to select a model with excellent face validity. Ischemic stroke accounts for 80% of all strokes, and the majority of these occur in the territory of the middle cerebral artery (MCA), often inducing infarcts that affect the sensorimotor cortex, causing persistent plegia or paresis on the contralateral side of the body. We demonstrate in this video a method for producing ischemic stroke in elderly rats, which causes sustained sensorimotor disability and substantial cortical infarcts. Specifically, we induce permanent distal middle cerebral artery occlusion (MCAO) in elderly female rats by using diathermy forceps to occlude a short segment of this artery. The carotid artery on the ipsilateral side to the lesion was then permanently occluded and the contralateral carotid artery was transiently occluded for 60 min. We measure the infarct size using structural T2-weighted magnetic resonance imaging (MRI) at 24 hr and 8 weeks after stroke. In this study, the mean infarct volume was 4.5% ± 2.0% (standard deviation) of the ipsilateral hemisphere at 24 hr (corrected for brain swelling using Gerriet’s equation, n = 5). This model is feasible and clinically relevant as it permits the induction of sustained sensorimotor deficits, which is important for the elucidation of pathophysiological mechanisms and novel treatments.

**Figure:**
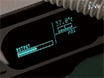


## Introduction

Stroke is currently the third most common cause of death worldwide and the leading cause of disability^1^. Ischemic stroke, which comprises 80% of all strokes, often results in infarcts in the cortex causing loss in sensation (*e.g.*, proprioception), in motor function and in attention to the affected side^2-4^. The middle cerebral artery (MCA) is the largest of the vessels that draws supply from the circle of Willis and stems from the internal carotid artery^5^. The MCA is the cerebral vessel most commonly affected in ischemic stroke, with strokes in this territory accounting for 65% of all ischemic strokes^6,7^. The MCA supplies both cortical and subcortical regions and neurological abnormalities caused by MCA stroke vary depending on the exact location of the occlusion^7^. Proximal MCA occlusions affect the deep territory through the lenticulostriatal arteries and cause large infarcts encompassing both cortical and subcortical regions. In contrast, more distal occlusions that deprive solely cortical regions of blood flow tend to produce smaller cortical infarcts.

In large population studies, human stroke lesions range from 5-14% of the ipsilateral hemisphere^8,9^; malignant stroke accounts for 10% of strokes and gives rise to larger infarcts, requiring a hemicraniectomy to reduce intracranial pressure, and patients with smaller lesions are more likely to survive^10^. We demonstrate a reproducible model that produces lesions that occupy a similar proportion of the hemisphere as many human strokes.

Stroke is a heterogeneous disease; 75% of ischemic strokes are induced by either lacunar infarcts (from obstruction of intracranial small vessels); cardioembolic stroke; or large artery atherosclerosis, which accounts for 30% of strokes. Symptomatic atherosclerosis is most frequently observed at the point where the common carotid artery (CCA) branches into the internal and external carotid arteries^11^.

Pre-clinical models of stroke should be as similar to the human condition as possible to simulate its pathophysiology and should incorporate the risk factors of stroke. 92% of ischemic stroke occurs in people over the age of 65, and other risk factors include obesity, high blood pressure, and atherosclerosis, as previously discussed^12^. To better represent these risk factors, it is recommended to use a model that may share some of the pathophysiological features of the natural condition. In this protocol, we have included advanced age and obstructed blood flow through the carotid arteries.

The classic model of middle cerebral artery occlusion (MCAO) is the intraluminal filament model of proximal MCA occlusion, which reduces blood flow in the anterior and middle cerebral arteries. Short occlusion times using this model focuses the lesion to the subcortical region, whereas longer occlusion times can result in large lesions recruiting areas of both the cortical and subcortical areas, resulting in a higher mortality rate in elderly rats. In comparison, the model used by our group involves performing a craniotomy and opening of the dura followed by coagulation of the blood and destruction of a small portion of the MCA using bipolar cauterizing forceps. This diathermy model is adapted from the 1981 paper by Tamura* et al.*^23^ and the use of the craniectomy may limit raised intracranial pressure, which is a feature of the closed skull, and results in higher reproducibility and a lower mortality rate in our surgery cohort compared to some other models^13^. To generate reproducible infarcts and sustained disability we permanently occlude the proximal CCA and transiently occlude the distal CCA as per Chen *et al.*^14^ We use non-invasive T2-weighted magnetic resonance imaging (MRI) to evaluate the extent and location of cerebral infarction, and the degree of brain swelling in the sensorimotor cortex.

## Protocol

This protocol was approved by Institutional guidelines set out by King’s College London, and was performed in accordance with the UK Home Office guidelines and Animals (Scientific Procedures) Act of 1986.  Guidelines may vary between institutions; please ensure adherence to institutional guidelines before attempting this procedure.  In order to maintain aseptic technique when touching equipment, autoclave a large piece of aluminium foil and use this to wrap around equipment handles such as on the microscope and anesthesia machine. Sterile cling film (plastic wrap) may also be used.

### 1. Preparation

1. Familiarize rats with gel fluid packs (veterinary recovery gel) and soft chow for at least 48 hr prior to stroke surgery. As animals often find difficulty with eating and drinking after the procedure, introduce the animals to these items before surgery, to minimize neophobia. House elderly rats with wooden chew blocks to reduce the incidence of overgrown teeth (See Section 6).
2. Sterilize all surgical tools before beginning surgical procedures by autoclaving (minimum 121 °C, 15 PSI, for 15 min). Sanitize all working surfaces using 1% chlorhexidine in 70% ethanol and use surgical drapes and maintain aseptic technique for the duration of the procedure.
3. Induce lesions on the hemisphere contralateral to the preferred paw. Allocate lesions to either the left or the right hemisphere depending on each rat’s preferred forepaw, determined by the pre-operative baselines in the Montoya staircase test^15^ (see **Figure 4**). For simplicity, when describing this procedure in this manuscript, the left paw is the preferred paw, and surgeries are performed on the right hemisphere. Use Lister Hooded or Long Evans rats for these behavioural tests as they learn quickly. Note: The staircase test is designed to measure changes in both fine and gross skilled movements following motor system damage. The staircase consists of seven steps on each side of a central platform. If equipment for the staircase test is unavailable, the cylinder test is a suitable alternative for testing handedness, but note that there is only a transient deficit on this test in this model of stroke, and therefore will be unsuitable to measure long-term recovery following treatment.
  1. Place three sugar pellets in the well of each step (21 pellets per side). Place rats in the staircase apparatus for 10 min and record the number of pellets retrieved and the number of pellets displaced on each side. Note: To be included in the task post-operatively, rats must retrieve a minimum of 75% pellets at baseline.

### 2. Surgery

1. Use female elderly Lister-Hooded rats at 16-18 months (250 to 400 g) and induce anesthesia with 5% isoflurane in 1.5 L/min O_2. _Following induction of anesthesia, reduce the level of isoflurane and maintain it at an adequate but minimized depth for surgery (*e.g.*, 1.5-2%). Deliver anesthesia to the animal by a face mask, and use a scavenging system to limit the surgeon’s exposure to isoflurane.
  1. Perform all procedures up to, but not including craniotomy on sham animals, as this procedure can produce behavioral deficits^16^. Consider experimental goals when designing MCAO experiments, and decide whether or not a sham group to control for craniotomy should be included.
2. Administer pain relief pre- or peri- operatively (Carprieve, 0.25 mg/kg, s. cut).
  1. Add 1 ml to 19 ml sterile 0.9% saline to make the stock solution and inject 0.6 ml per 300 g rat. The use of Carprieve is preferred over Carprofen because the former is stable at RT.
3. Shave the fur on the ventral neck region and temporal region on the right hemisphere to expose the skin. Disinfect the surgical sites using ethanol swabs. Be sure to follow local IACUC guidelines for hair removal. 
  1. Perform this step away from the operating area to minimize the amount of fur at the incision site.
4. Place the rat on a corkboard covered with a sterile drape in the supine position on a heating pad. Apply lidocaine cream to the shaved regions of the head and neck. Insert a rectal probe to monitor and maintain the animal’s temperature between 36.5-37.5 °C with a homeothermic system.
  1. Put lacrilube ointment on the eyes to prevent drying. Give the rat an injection of atropine sulphate solution (0.05 ml of a 600 µg/ml solution, subcutaneously) to reduce tracheal secretions. Note: Researchers should consider measuring physiological variables such as blood gases and pressure.
5. Before beginning surgery, check the hindpaw pinch withdrawal and blink reflexes to confirm full anesthesia.
6. Under a dissecting microscope, make a 2 cm central midline incision on the exposed neck using a scalpel. Move the salivary glands gently lateral to the trachea on both sides.
7. Loop non-absorbable silk suture around the skin that overlies the common carotid artery on one side. Gently pull away the skin from the site using the silk suture and stick these down to the corkboard using surgical tape to reveal the common carotid artery. Carefully blunt dissect the arteries free from surrounding fascia and the vagal nerves using fine forceps.
  1. Be careful not to damage the muscle or vagus nerves during this step, as this will impair feeding, swallowing and breathing.
8. Once the common carotid artery is exposed, use non-absorbable silk suture (5/0) to isolate the carotid, first separating the carotid from the vagus nerve by reverse dissecting with fine forceps and being careful not to make contact with the nerve. Once the vessel has been looped (but not tied) with the suture, tape the ends of the suture together using surgical tape to prevent this step being undone. Remove the sutures holding back the overlying skin.
9. Repeat for the other side (Sections 2.7-2.8)
10. Place a sterile saline-soaked gauze into the wound to keep tissues moist during the remainder of the surgery. Loosely suture the skin and place an additional saline-soaked gauze over the area to prevent further dehydration.
11. Place rats in a lateral position and make an incision in the skin at the midpoint between the right orbit and the external auditory canal. Retract the skin using up to five elastic 3 mm hook retractors pinned to the corkboard and then blunt dissect the temporalis muscle to reveal the skull.
12. Place a thumb wheel-adjusted gravity-driven saline drip above the open site (flow rate approximately 2 ml/min) and set up an aspirator system to remove bone debris and to clear minor bleeding from the exposed site throughout this phase of the surgery (Sections 2.13-2.16).
  1. Place the saline delivery nozzle at the highest point of the skull near the ear, and the aspirator nozzle at the lowest point. Adjust the wheel during bleeds to supply more saline to the area to better visualize the source of any bleed for cautery of the vessel, reducing the amount of time the animals are under anesthesia.

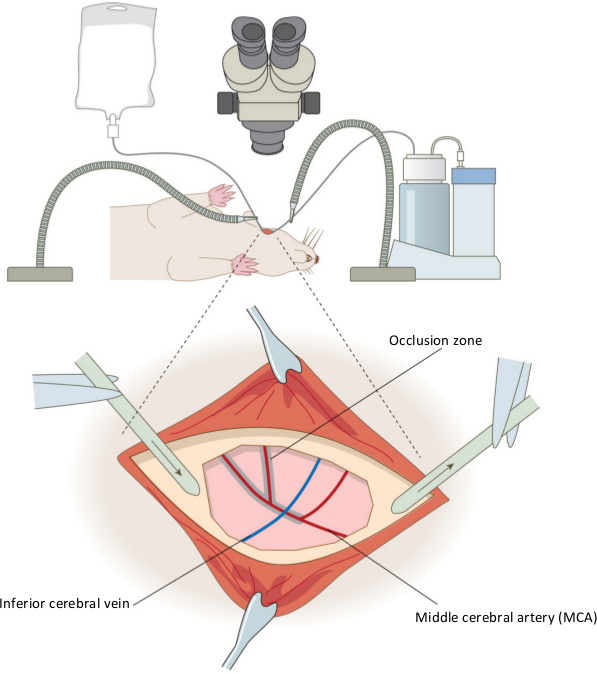
**Figure 1. Surgical set up of the permanent distal middle cerebral artery occlusion model.** The equipment used in the set up for the rat craniotomy is shown for the right hemisphere, and inset, the positioning of the aspirator and saline drip around the craniectomy site. Also shown are the key features of the vasculature; the middle cerebral artery (red) and inferior cerebral vein (blue) are shown, and the shaded area indicates where coagulation of the artery occurs. Confirmation of the occlusion is performed by cutting the MCA below the inferior cerebral vein. Please click here to view a larger version of this figure.

1. Perform a craniotomy on the exposed region (approximately 5 mm x 5 mm) using a dental drill with a coarse 1.6 mm diamond coated drill burr at approximately 8,000 rpm, ensuring the application of circular and lateral pressure and not downward pressure whilst drilling the exposed area. Once the bone is thin enough that it looks completely transparent, remove using forceps.
2. Use a homemade dural hook, made by bending one tip of fine forceps approximately 180° to form an arc, to carefully open the dura, being cautious to avoid the large surface blood vessels, as they are delicate and easy to rupture. Note: The exposed area of the brain will reveal the middle cerebral artery (MCA); the desired segment measures approximately 2 mm in length (see **Figure 1**, inset).
3. Coagulate the MCA from where the inferior cerebral vein crosses, to the point of artery bifurcation and then along the caudal branches of the MCA using a pair of diathermy forceps until fully occluded^17^. Use angled Jeweler diathermy forceps with 0.25 mm pointed tips.
  1. When occluding the artery, alter the saline flow as necessary to keep this area cool, preventing the coagulating forceps from adhering to the blood vessel. When occluded, the blood vessel appears black and no sign of blood flow should be present; blood flow can be seen in partially occluded vessels.
  2. Cut the MCA at this point to confirm complete occlusion. Using microvascular scissors, cut beneath where the inferior cerebral vein crosses the MCA.
  3. Cover the exposed area with a saline-soaked gauze pad before continuing to the next step.
4. Turn the rat back to a supine position and reopen the loosely tied suture on the neck to re-expose the carotid arteries. Ligate the carotid artery on the same side as the occluded MCA (right) permanently by tying a knot in the silk suture around the carotid, whilst the left carotid artery is occluded transiently using a 13 mm stainless steel artery clip with 125 g pressure for 60 min. Loosely suture up the incision in the neck during this time and place a sterile saline soaked gauze on top to prevent dehydration.

1. Note that the animal’s right hand side will be on the surgeon’s left hand side, given the supine positioning.
2. For sham animals, open the ventral part of the neck and temporal regions and separate the muscles to locate the common carotid arteries, but do not occlude. Have sham animals undergo procedures up to but not including craniotomy, as this procedure can produce behavioral deficits^16^. To maintain blinding of other team members (*e.g.*, during later behavioral testing), perform incisions and suturing.

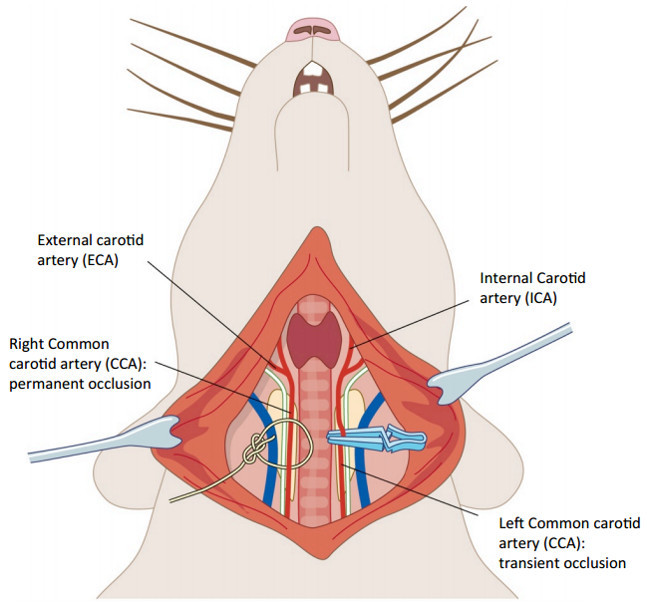
**Figure 2. Tandem carotid occlusion following middle cerebral artery occlusion. **The right common carotid artery (CCA) is permanently occluded by tying a silk suture (5/0) around the blood vessel (on the left hand side of the image). The left CCA (on the right side) is occluded for 1 hr using a microvascular clamp. These surgeries were performed taking care not to make contact with the vagus nerve on each side (white). Please click here to view a larger version of this figure.

1. Suture the incision in the temporal region during the 1 hr of left common carotid occlusion.
2. Give rats 20 ml of saline subcutaneously (5 ml to two sites on each flank) to maintain hydration (see 6.2).
3. When 60 min has passed, remove the clip. Apply saline topically to the surrounding muscle, and close the incision in the neck using subcutaneous continuous stitching of absorbable sutures (4/0).

### 3. Post Operative Care

1. Place the rat in a 31 °C incubator to recover from anesthesia for up to 4 hr. Note: Researchers may prefer to use other temperatures and durations according to local practice.
2. Repeat carprieve (0.25 mg/kg), for pain relief at 24 hr. Because all animals receive the same dose of Carprieve, this factor is controlled for systematically. Any neuroprotective effect of Carprieve is likely to be negligible.
3. Give saline injections daily to prevent dehydration for at least the first three days. On the assumption that a rat requires 65 mL fluid per kg body weight (each 24 hr), ensure that each 300 g rat receives ~22 ml fluids per day.

### 4. Confirmation of Infarct

1. To measure the infarct volume use structural MRI. Note: An alternative method could be to use histological staining, such as tetrazolium chloride (TTC), which has been previously correlated to structural MRI data^18^. However, this can only be used at the end point of the study, and not longitudinally.
  1. Twenty-four hours after the induction of MCAO, anesthetize rats with isoflurane (5% for induction, 1-1.5% for maintenance) in 0.9 L/min Medical Air and 1 L/min O_2_, secure in a quadrature birdcage magnetic resonance coil (43 mm diameter) and place in a 7 Tesla horizontal bore scanner.
  2. Obtain T2 weighted scans using a fast-spin echo sequence: echo time (TE) 60 msec, repetition time (TR) 4,000 msec, field of view (FOV) 40 x 40 mm, acquisition matrix 128 x 128, acquiring 40 x 0.5 mm thick slices in approximately 8 min. Subsequently, convert 40 slices into 20 x 1 mm thick slices by using the resampling function in medical imaging software.
2. Obtain lesion volumes in a medical image display package by measuring the cross-sectional area of infarct in 20 volumes. Obtain the total volume by multiplying the sum of these areas by the thickness (1 mm). Calculate the group mean volume and standard deviation. For percentage lesion volume calculations, also acquire the volumes of the ipsilesional and contralesional hemispheres.
3. To correct for brain swelling due to edema, adjust these values using Gerriets’ formulae^18^. Only include slices that contain cortex and do not contain the cerebellum or olfactory bulbs according to a standard rat atlas^19^ to avoid over-correction, as this can generate negative lesion volumes.
  1. Acquire T2 weighted images again 8 weeks after stroke surgery.

### 5. Sample Size Calculations for Future Studies Assessing Neuroprotection and Behavioral Recovery

1. Perform sample size calculations to estimate the minimum sample size that would be required in future hypothetical neuroprotective experiments using two groups (control vs*.* treatment) to identify treatment effects of three different magnitudes (25% reduction, 50% reduction, 75% reduction) using the *a priori* algorithms implemented in power analysis software^20^. Note: To enable readers to do similar calculations with their own data, there are screenshots from freely-available power analysis software (see Representative Results). Use the following parameters: acceptable false positive rate (*i.e.*, type I error threshold; α) ≤0.05, and power (which equals 1-β) ≥0.80 (*i.e.*, greater than 80% power). See below for explanations and a discussion^21^.

### 6. Elderly Animal Welfare After Stroke Surgery

1. Make available additional soft chow and rehydration gel packs that the rats were habituated to before surgery, in addition to water bottles with extended tips for easier reaching. In addition, have an absorbable recovery pad in the cages instead of loose bedding (*i.e.*, avoid wood chip) for the first 24 hr, and provide extra nesting bedding. Do not use pureed food (*e.g.*, baby food) as rats with dysphagia may choke.
2. Weigh animals daily for 7 days to monitor recovery. Weight loss is a primary indication of dehydration and stress. Weight loss in the first few days after surgery principally reflects dehydration (rather than loss of body weight due to reduced feeding).
3. Where an ageing rat shows weight loss unrelated to surgery, replace lost body weight with an equivalent weight of fluids and inspect the top and bottom teeth. Where teeth are overgrown, anesthetize the rat with isoflurane (as in section 2.1), and place in a supine position
  1. Place a 1 ml syringe barrel behind the teeth to protect the soft tissues. Cut using a hand-held circular saw (*e.g.*, approximately 3 cm diameter) at high rotary speed but with slow, firm hand movements (*e.g.*, 3 sec cuts). Allow the rat’s teeth to cool down between cuts. Ensure that the rotary saw is mounted on the mandrel so that the saw’s teeth face in the direction of rotation. It is worth keeping individually wrapped, sterilized rotary saws ready for such eventualities. Note: Overgrowth can occur in elderly rats even when maintained on a hard pellet diet. We recommend that teeth are checked in elderly rats regularly.
4. Where an elderly rat shows weight loss and piloerection unrelated to surgery, discuss treatment options with a veterinarian. Consider humanely killing the animal. MRI of such animals can show a pituitary tumor (often in elderly female rats) that is inoperable and fatal.

## Representative Results

Permanent MCAO was induced by performing craniotomy, followed by coagulation and destruction of the middle cerebral artery by diathermy combined with permanent occlusion of the ipsilesional common carotid artery and 60 min occlusion of the contralesional common carotid artery. A schematic of the setup of the equipment and occluded MCA is shown in **Figure 1**, and of the carotid arteries in **Figure 2** (above)**.**

Stroke outcome was assessed 24 hr and 8 weeks after stroke by measuring the infarct volume on 40 x 0.5 mm slices (from rostral end of olfactory bulb to rostral end of spinal cord) using the Region of Interest toolkit in a medical image display package. A representative T2 weighted structural MRI scan is shown for the same animal at 24 hr and 8 weeks (**Figure 3A**). The infarct volume was identified by the areas of the rat brain showing a hyperintense signal; as T2 weighted images show water or plasma as a bright white area. It is known that there is an increase in edema and brain swelling after stroke, and this can be measured from a T2-weighted scan which has been correlated to histological measurements of infarct volume^18^. However, edema present early after stroke (*e.g.*, at 24 hr) can lead to an overestimation of the final lesion volume (*e.g.*, at 8 weeks) and therefore we also present mean infarct volumes adjusted using Gerriet’s formulae. **Figure 3B** shows mean data from the raw (unadjusted) lesion volume at 24 hr as 62.8 mm^3^ (± 25.4 mm^3^ SD, top graph); this occupies 9.8% of the affected hemisphere (± 4.2% SD, middle graph). When corrected for brain swelling using Gerriets’ formulae this value is reduced to 4.5% (± 2.0% SD, bottom graph).

Stroke severity was also measured using the Montoya staircase test^15^. In brief, animals were pre-trained to retrieve sugar pellets for 4 weeks prior to MCAO stroke surgery, and tested for 8 weeks following stroke (**Figure 4**) to confirm a sustained deficit. Rats were placed in the staircase apparatus for 10 min and the number of pellets retrieved was recorded (out of 21 pellets) and displayed as a percentage (group means ± standard errors). A regression analysis was performed to fit the line to the data.

**Figure 5** shows the sample size calculation using infarct volume data (for potential effects of candidate therapies), analyzed using an algorithm in power analysis software for a t-test using “Difference between two independent means (two groups)” and using the (uncorrected) means and standard deviations from **Figure 3B**. The information in **Figure 5** and **Table 1 **show that 12 rats would be required per group to detect a therapy that reduced infarct volume by 50% at 24 hr, whilst **Figure 6** shows an “X-Y plot” of power achieved using varying numbers of animals. **Table 1 **summarizes sample size calculations for all time points.


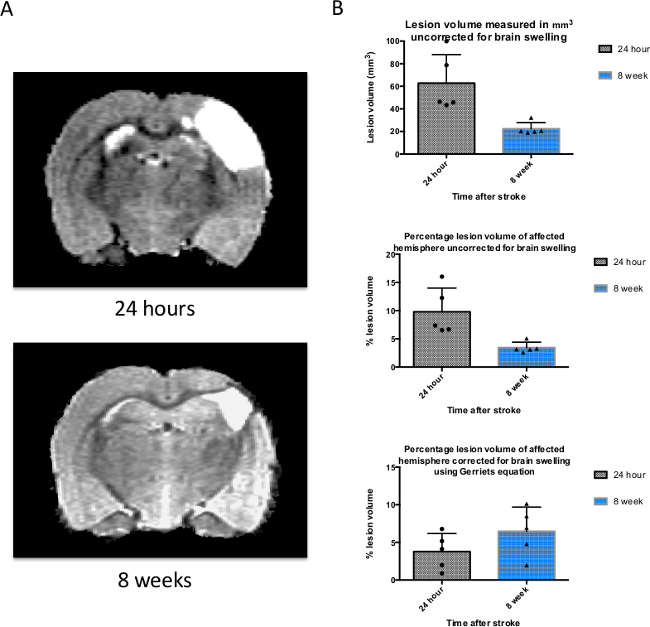
**Figure 3. T2-weighted structural MRI is used to measure the size of the infarct and brain swelling after stroke. **(**A**) A T2-weighted magnetic resonance image of the same rat brain 24 hr and 8 weeks after induction of stroke. The white area represents the lesion, but also contains some vasogenic edema that resolves by 8 weeks. (**B**) Infarct volumes were measured using a medical image display package Region of Interest Toolkit, and are plotted on a graph representing the mean ± SD for the 3 time points used (n = 6). Raw lesion volume (not corrected for brain swelling due to edema), percentage lesion of affected hemisphere (uncorrected for swelling), and percentage lesion of hemisphere corrected for brain swelling using Gerriets’ formulae are shown here. SD was used rather than SEM in order to perform sample size calculations (see **Figure 5**). Please click here to view a larger version of this figure.


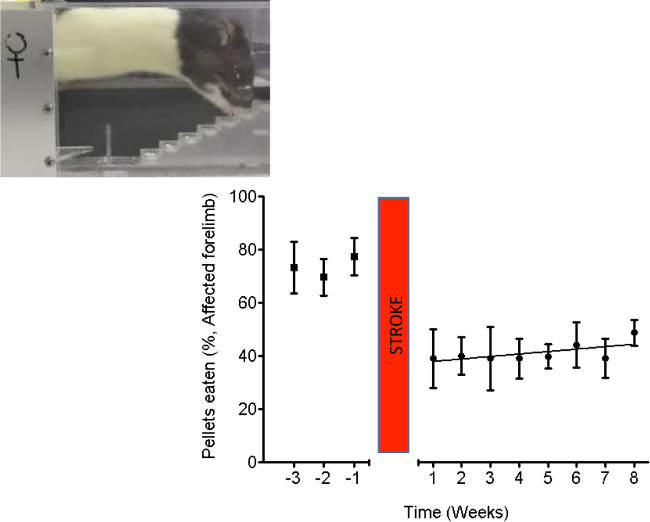
**Figure 4. The staircase test shows impairments in grasping and retrieving pellets. **In this stroke model there was very little spontaneous recovery. Stroke in elderly rats persistently impairs dexterity, shown by weekly testing using the “staircase test” of pellet reaching. Inset: A picture of a rat performing the behavioral test. The graph shows the mean (± standard error) number of pellets retrieved (out of 21, expressed as a percentage) per week by the affected forepaw. n = 5. Please click here to view a larger version of this figure.


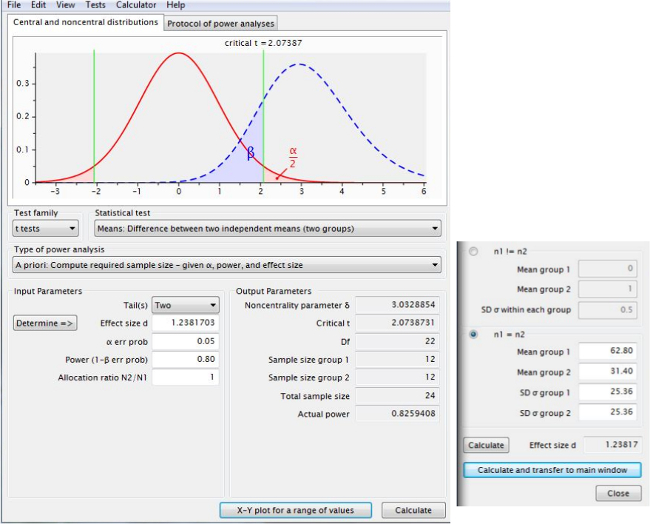
**Figure 5. Sample size calculations to determine group numbers of rats required to detect a desired therapeutic effect.** This screen shot, taken from power analysis software, shows that 12 rats per group would be required to detect a therapy that reduced infarct volume by 50% at 24 hr. Please click here to view a larger version of this figure.


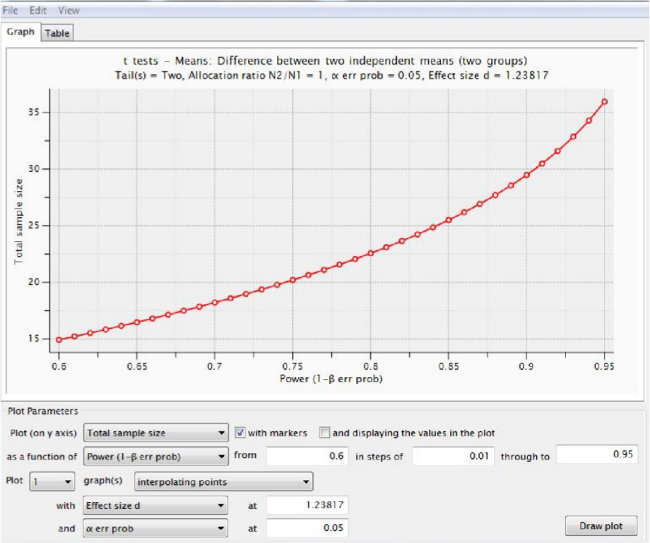
**Figure 6. Power achieved using various total numbers of animals.** An “X-Y plot for a range of values” from power analysis software shows the power that would be obtained for experiments using various (total) numbers of elderly rats, given the parameters shown in **Figure 5**. **Table 1** summarizes all our results. Please click here to view a larger version of this figure.

**Table:** 

**Time after stroke:**	**Number of rats per group required to detect a reduction in lesion volume of:**
75%	50%	25%
24 hr	6	12	42
8 weeks	4	5	17

**Table 1. Calculations of sample sizes per group for hypothetical future experiments.** Calculated using power analysis software (See **Figures 5** and **6**). Table shows number of rats per group required for a two-group experiment to detect a 25%, 50% and 75% reduction in lesion volume at each of the timepoints in this study.                                                      

## Discussion

MCAO in rodents is a technique often used to model human stroke. This model does have a few details to note in the protocol. Firstly, it is essential to maintain the animal’s body temperature throughout the experiment as this affects the size of the infarct and the number of mortalities in a study. It may be possible to discontinue isoflurane during the transient occlusion of the right CCA and keep rats in a warmed, quiet environment to increase survival rates by reducing exposure to isoflurane. Researchers should consider whether a shorter anesthesia period outweighs the stress of additional induction. The vasculature (*e.g.*, MCA branching) of the rats varies within and between cohorts of animals^22^. It is important to bear this in mind when beginning a new study. Different CCA occlusion times can be evaluated (*e.g.*, 30, 45, 60 and 90 min). In this study an occlusion time of 60 min is used. In other studies we have found that 45 min occlusions cause a similar sized cortical infarct but with anecdotal evidence of improved survival rates. Accordingly, have the surgeons start with a short occlusion time (*e.g.*, 30 min) to see whether adequate lesion volumes (and/or required behavioral deficits) are obtained and only then to increase occlusion times where necessary. Behavioural deficits are not sustained in adult rats compared to elderly rats with identical occlusion times.

MRI can be used to judge whether (after a particular occlusion time) lesion volumes are appropriate for the study goals. A small lesion would span less than ten 0.5 mm coronal slices (out of 40). A medium size lesion would span between ten and twenty coronal slices. A large lesion would span between twenty and thirty coronal slices. A very large lesion would span more than thirty of the forty slices. In our experience, rats with very large lesions (more than thirty slices) and/or evidence of herniation across the midline usually have poor prognosis: shorter occlusion times might be considered. MRI is also useful for assessing the infarct location: some are more caudally located and some are more rostrally located.

Take extra care when separating the vagus nerve from both common carotid arteries. Rales (rasping) may occur after stroke surgery and this could be due to nerve damage in some animals, although the cause is currently unclear: in our experience, prognosis is very poor for these animals and it is usually recommended to humanely kill them.

The permanent diathermy MCAO model results in reproducible cortical infarcts and acceptable post-operative survival rates in elderly rats. The technique does, however, require invasive surgery under a stereomicroscope. It is important to maintain aseptic technique if animals are to recover well from surgery. Care must be taken not to damage the MCA while exposing and coagulating the artery, and damage to the cortical surface should be minimized otherwise the exposed area of the cortex may form part of the infarct area. It is recommended to obtain as much experience as possible to establish the procedure and to achieve consistent infarcts and determine occlusion times before a study is performed to test candidate therapies, for example. Experimenters should randomize any treatments within surgery sessions (“block randomization”) where possible. It is worth noting that this model does not involve MCA reperfusion (unless transient MCA ligation is used instead of diathermy). Mortality can be high in elderly rats with these large cortical infarcts but it should be possible to reduce mortality by using shorter occlusion times and by minimizing exposure to general anesthesia where possible (*e.g.*, during occlusion). The use of 70% N_2_O and 30% O_2_ as a carrier may allow lower levels of isoflurane to be used: this reduced exposure to isoflurane may result in higher survival rates.

Another point to consider is that atherosclerosis is a gradual process, whereas in this protocol we simulate it with acute CCA occlusion. However, the substantial reduction in blood flow and sustained deficits simulate tandem occlusions that occur in many stroke patients. Permanent distal MCAO without tandem CCA occlusion in rats fails to induce strokes reproducibly^14^: moreover, without tandem CCA occlusion, we have found considerable spontaneous recovery occurs that precludes long-term behavioral evaluation of stroke therapies over 8 weeks. In contrast, we show that distal MCAO with tandem CCA occlusion induces long-term deficits in elderly rats.

In conclusion, this procedure in rats causes strokes that are similar in size and location to those seen in the human condition, with sustained disability that one can use to enable the testing of novel treatments and elucidation of repair mechanisms after ischemic stroke.

## Disclosures

The authors have nothing to disclose.
